# AmpliFuse: an amplicon simulation tool with enhanced chimera generation for Illumina platforms

**DOI:** 10.1128/mra.01439-25

**Published:** 2026-01-27

**Authors:** Mingsong Kang

**Affiliations:** 1 Ottawa Laboratory–Fallowfield, Canadian Food Inspection Agency 5737, Nepean, Ontario, Canada; University of Michigan, Ann Arbor, Michigan, USA

**Keywords:** amplicon sequencing, chimera formation, simulator, benchmarking

## Abstract

PCR-generated chimeric amplicons undermine the accuracy and reliability of subsequent amplicon-sequencing analyses. AmpliFuse, a Python-based tool, simulates realistic amplicon datasets through *in silico* PCR, chimera formation, and read simulation. By providing biologically relevant chimera-containing amplicon reads, AmpliFuse would facilitate benchmarking of chimera-detection algorithms and workflows, advancing microbiome and AMR research.

## ANNOUNCEMENT

Polymerase chain reaction (PCR) amplification can potentially generate artificial recombinants when incomplete extension products serve as primers for heterologous templates (1). These chimeric amplicons compromise the accuracy of richness estimates and introduce erroneous associations, particularly in the contexts of amplicon-based microbial community profiling and antimicrobial resistance (AMR) surveillance (2–4). Consequently, removing chimeras is an essential step in analyzing amplicon sequencing data (5). While empirical datasets generated from defined mock-community experiments capture genuine error structures, their inherent complexity frequently complicates the validation of chimera-detection algorithms. Existing bioinformatic tools that generate chimera-containing datasets for benchmarking purposes usually lack support for multiple primer pairs and updated sequencing error models across platforms (Table 1). Additionally, current read simulators typically focus on modeling substitution or indel errors but do not integrate modules for chimera formation (Table 1). To address these limitations, AmpliFuse was developed to generate Illumina amplicon reads with realistic chimera formation and sequencing errors, providing an ultimate resource for benchmarking and workflow evaluation.

**TABLE 1:** Characteristics of chimera formation and the short-read simulators

Type	Name	Output^*a*^	Amplicon sequencing	Primer pairs	Designated primer	Sequencing error model	Chimera algorithm	Reference
Chimera formation and short-read simulator	Amplifuse	PE fastq	Yes	Multiple	Yes	HiSeq; miSeq; NextSeq; NovaSeq	Simera 2-based	This study
	Grinder	Fastq;fasta	Yes	Single	Yes	Illumina; 454; Sanger	*k-mer*-guided breakpoint; random breakpoint	(6)
Chimera formation	Simera	fasta	Yes	Single	Yes	na^*b*^	Simera 1 and 2	(7)
Short-read simulator	ISS	PE fastq	Yes	No	na	HiSeq; miSeq; NextSeq; NovaSeq	na	(8)
	Art_modern	PE, SE fastq	No	na	na	HiSeq; MiSeq MiniSeq; NextSeq	na	(9); https://github.com/YU-Zhejian/art_modern
	DWGSIM	PE, SE fastq	No	na	na	User-defined rate	na	https://github.com/nh13/DWGSIM
	Mason	PE, SE fastq	No	na	na	Illumina; 454; Sanger	na	(10)
	NEAT	PE, SE fastq	No	na	na	Illumina	na	(11)
	wgsim	PE, SE fastq	No	na	na	User-defined rate	na	(12)

^*a*^ PE, pair-end; SE, single-end.

^*b*^ na, not applicable.

**Fig 1:**
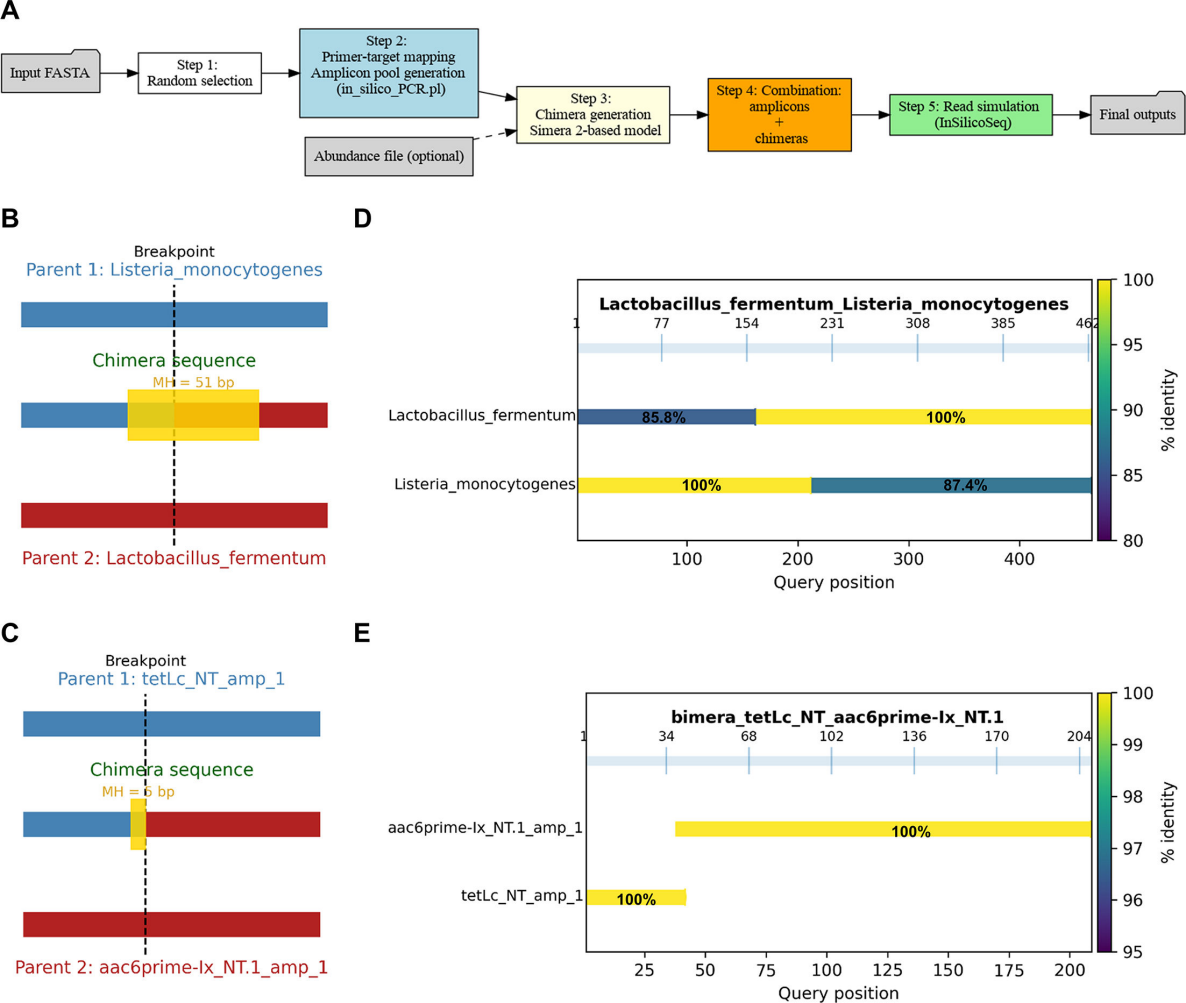
AmpliFuse workflow and representative chimera formations with alignment analyses. (**A**) Overview of the AmpliFuse workflow. (**B and C**) Schematic of representative chimera formations using datasets derived from ZymoBIOMICS 16S V3–V4 mock community (**B**) and the AMR panel (**C**). The expected breakpoint (dashed line) lies within a microhomology (MH) highlighted in yellow. (**D and E**) Example alignments of two parent amplicons to a representative chimeric amplicon generated from the ZymoBIOMICS (**D**) and AMR panel (**E**) datasets, respectively. Under the default settings, chimeric amplicons account for approximately 15.4 and 4.9% of all amplicons generated using the ZymoBIOMICS and the AMR panel datasets, respectively.

AmpliFuse was developed in Python v3.13.7 with NumPy v2.3.3 and Biopython v1.85 on Ubuntu 22.04 using PyCharm 2025.2.4 as the development environment. GitHub Copilot v1.5.59-243 (GPT-5 mini) was used to assist with code debugging and incremental refactoring to improve readability. AmpliFuse has also been successfully tested on Ubuntu 24.04 and macOS Tahoe 26.1. In the current version, AmpliFuse comprises a series of steps to ensure effective operation (Fig. 1A). Initially, a subset of template sequences randomly selected from an input FASTA file is used to define the amplicon pool via *in_silico_PCR.pl* v 0.6 (https://github.com/egonozer/in_silico_pcr). Subsequently, the generated amplicon FASTA file, together with an optional user-provided abundance file, serves as input for chimera formation using the Simera 2-based model (7) with a stochastic PCR branching process. In this model, chimera formation is weighted by microhomology length, parent-pair abundances, and the fragment-formation probability, which accounts for factors, such as the number of PCR cycles and amplicon length. Finally, the combined parent and chimera amplicons are processed with *InSilicoSeq* v2.0.1 (8) for Illumina read simulation using the amplicon model with configurable read counts and sequencing error models.

AmpliFuse was demonstrated utilizing two publicly available datasets, namely, ZymoBIOMICS Microbial Community (https://zymoresearch.eu/collections/zymobiomics-microbial-community-standards/products/zymobiomics-microbial-community-standard) using 16S rRNA gene V3–V4 primer pairs (341F and 805R) (13) and the Illumina AmpliSeq AMR Research Panel (the AMR panel) (https://www.illumina.com/products/by-brand/ampliseq/community-panels/antimicrobial-resistance.html). During demonstration runs with default settings, AmpliFuse successfully generated a diverse array of recombinant chimeras. Representative chimeric amplicons from both datasets (Fig. 1B and C) exhibited strong concordance with alignment-based analyses (Fig. 1D and E), confirming the accurate generation of chimeric sequences in accordance with the defined parameters.

AmpliFuse facilitates the generation of synthetic chimera-containing datasets, which are well-suited for evaluating chimera-removal algorithms and assessing the robustness of classifiers in high-diversity microbial communities. Additionally, AmpliFuse may serve as a practical tool to support primer design by enabling the comparison of candidate primer sets and prioritizing those least likely to produce non-specific or chimeric amplicons, before laboratory validation.

## Data Availability

AmpliFuse is accessible through https://github.com/comingkms/AmpliFuse (GPL v.30) and archived on Zenodo (DOI: https://doi.org/10.5281/zenodo.17859476).

## EDITOR'S NOTE

**Ed. Note**: This article was handled by Pat Schloss who acted as a guest editor in consultation with the Editor-in-Chief, Irene Newton. Dr. Schloss's role as the guest editor of this article is outside of his duties as the chair of the Journals Committee.
